# Chronic Kidney Disease Requiring Hemodialysis as a Significant Predictor of Target Lesion Revascularization After Endovascular Treatment of Femoropopliteal Occlusive Lesions with a Drug-Coated Balloon

**DOI:** 10.3390/jcm14051474

**Published:** 2025-02-22

**Authors:** Tsuyoshi Ichinose, Toshifumi Kudo, Yohei Yamamoto

**Affiliations:** 1 Vascular Surgery, Institute of Science Tokyo Hospital, Tokyo 113-8519, Japan; t-kudo.srg1@tmd.ac.jp (T.K.); y-yamamoto.srg1@tmd.ac.jp (Y.Y.); 2 Vascular Surgery, IMS Tokyo Katsushika General Hospital, Tokyo 124-0025, Japan

**Keywords:** drug-coated balloon, endovascular therapy, femoropopliteal, chronic kidney disease, hemodialysis

## Abstract

**Background:** Drug-coated balloons (DCBs) have been reported to have lowered the rate of restenosis and revascularization after endovascular treatment (EVT) of femoropopliteal (FP) lesions. Meanwhile, chronic kidney disease requiring hemodialysis (HD), which is becoming more prevalent in Japanese clinical settings, has been associated with poorer outcomes after EVT for FP lesions. This study aimed to retrospectively analyze the impact of HD on the outcomes of EVT using a DCB in a single center. **Methods**: This study included 161 consecutive FP lesions in 127 patients treated with a DCB between September 2018 and May 2023, stratified into HD (34.6%) and non-HD (65.4%) groups. The primary endpoint was clinically driven target lesion revascularization (CDTLR), and the secondary endpoints were major amputation and all-cause mortality. **Results**: The median observation period after EVT using a DCB was 336 days. Although a Rutherford’s category of 4 or higher was significantly predominant in the HD group (82.3%) than the non-HD group (53.5%), a Rutherford’s category of 4 or higher itself was not a statistically significant factor of the primary endpoint. The ratio of occluded lesion was significantly higher in the non-HD group (21.2%) than the HD group (8.1%). The duration of freedom from clinically driven target lesion revascularization (CDTLR) assessed via the Kaplan–Meier method was significantly shorter in the HD group (744 days) compared to the non-HD group (1533 days). The HD group had a higher incidence of CDTLR (odds ratio 4.48, *p* = 0.03) compared to the non-HD group. **Conclusions**: HD patients had significantly worse prognoses in EVT of FP lesions using a DCB.

## 1. Introduction

Endovascular treatment (EVT) using a drug-coated balloon (DCB) for lower extremity arterial disease (LEAD) offers higher patency rates than balloon dilatation without a drug coating in lesions involving the femoropopliteal (FP) arteries [1,2]. Beyond improving patency, DCBs represent a preferred modality for the revascularization of FP arterial lesions due to their “leave nothing behind” strategy which makes subsequent therapeutic options feasible [3]. LEAD patients commonly have multiple vascular risks. In Japan, chronic kidney disease (CKD) requiring hemodialysis (HD) is highly prevalent, with approximately 20–40% of patients treated with drug-coated balloons (DCBs) for femoropopliteal (FP) lesions in lower extremity artery disease (LEAD) on regular HD [4,5,6], reflecting a national prevalence of 1 HD patient per 359 individuals [7]. This high prevalence of HD patients makes Japan an appropriate setting for studying the impact of hemodialysis on FP EVT. Balloon angioplasty without a drug coating has been reported to result in worse outcomes for HD patients [8]. In HD patients, DCBs are reported to provide better outcomes for FP EVT compared to plain balloons [9]. However, major studies of drug-coated balloons for LEAD often exclude patients on HD [2,10,11,12] and the prediction of outcomes for the HD cohort of LEAD patients after the treatment with a DCB is difficult. To address this gap, this study aimed to examine the real-world impact of HD on outcomes of the FP EVT using DCBs at a Japanese institution.

## 2. Materials and Methods

The EVT records from September 2018 to May 2023 were retrospectively reviewed. All FP lesions treated using DCBs were included. The indication for EVT was category 2 or more based on the Rutherford classification. During each session, DCBs were utilized at the discretion of the respective physician, limited to cases with Peripheral Arterial Calcium Scoring System (PACSS) grades 1 or 2 or lower [13]. DCBs were used after the pre-dilation in all cases. No target lesions were treated with atherectomy devices, as atherectomy devices were not reimbursed in Japan during this study period. Bail-out stenting was not observed in this study cohort. The inflation time of the DCB to treat the target FP lesions was 3 min. The patient’s age, sex, and vascular risks (diabetes mellitus [DM], CKD, CKD on HD, smoking status, hypertension [HTN], and dyslipidemia [DL]) were collected from the medical records. In terms of LEAD severity, the patients with intermittent claudication were classified under Rutherford’s category 2 to 3, while those with chronic limb-threatening ischemia were classified under groups 4 to 6. The lengths of the lesions were measured based on angiography at the EVT session. Severely calcified lesions with a PACSS of 3 and 4, as well as long obstruction lesions corresponding to TransAtlantic Inter-Society Consensus II Type D, were not treated with DCBs in this cohort. The outflow below the knee lesions of the target lesion was not examined in this study. The patients underwent follow-up with ultrasonography and ankle branchial index the next day and every three months thereafter. After the first year, patients were followed up every three or six months, depending on each patient’s clinical course.

The primary outcome was clinically driven target lesion revascularization (CDTLR). Amputation of the limb of the target lesion and all-cause mortality were also surveyed as the secondary outcomes. Amputation was defined as a lower limb amputation at a level proximal to the ankle joint, with toe and foot amputations excluded. The outcomes were evaluated until May 2023. Differences in patient demographics between patients on HD (HD group) and patients not on HD (non-HD group) were analyzed using Fisher’s exact test for binary variables (DM, CKD, CKD on HD, smoking status, HTN, DL) and sex. The differences in patients’ age, observation period, and lesion length between the HD group and the non-HD group were assessed using the Wilcoxon rank-sum test, as these variables exhibited a non-normal distribution. Time-to-event, which included the freedom from CDTLR and amputation-free survival in this study, stratified by the presence of HD-requiring CKD, was analyzed using the Kaplan–Meier method. The statistical significance of differences in the duration of freedom from CDTLR and amputation-free survival between the HD and non-HD groups were examined by means of log-rank test. The relationship between the duration of freedom from CDTLR (primary outcome) and patient demographics was analyzed using multivariate Cox regression. Additionally, the relationship between amputation-free survival (secondary outcome) and patient demographics was assessed using the same method. A *p* value of less than 0.05 was considered as statistically significant. Statistical analyses were performed using EZR (version 1.64; Saitama Medical Center, Jichi Medical University, Saitama, Japan), which is a graphical user interface for R (version 4.3.1; The R Foundation for Statistical Computing, Vienna, Austria) [14].

## 3. Results

### 3.1. Patients and Lesions

A total of 161 lesions in 127 patients were identified as femoropopliteal lesions treated with a DCB and were included in this study. The average observation period was 409.9 days (range, 1–1577 days). Among the 127 patients included in this study, 44 (34.6%) were on HD (HD group) and 83 (65.4%) were not on HD (non-HD group). The characteristics of the patients in both groups are summarized in Table 1. CDTLR was observed in 16 lesions in the HD group and 10 lesions in the non-HD group, with a significantly higher incidence in the HD group (*p* = 0.01). One patient from each group underwent a major limb amputation (*p* = 1). The median observation period after the EVT was 336 days (interquartile range [IQR] 152–614). The median age of patients in the HD group was 72.5 years (IQR 64.0–77.5), which was significantly lower than that of the non-HD group (median 76.0 years, IQR 70.5–82.0; *p* < 0.001). The prevalence of HT, DL, DM, and smoking history was not significantly different between the two groups. Coronary artery disease (CAD) was significantly more prevalent in the HD group (*p* < 0.001), while the prevalence of cerebrovascular disease was not significantly different between both groups. The characteristics of the lesions of the two groups and the utilized DCBs are also summarized in Table 1. Rutherford’s category 2 and 3 lesions were more prevalent in the non-HD group. In contrast, category 4, 5, and 6 lesions were more common in the HD group (*p* < 0.001). Occlusions were more common in the non-HD group (*p* = 0.05). Calcifications were significantly more prevalent in the HD group (87.1% vs. 68.9%, *p* = 0.01). The average lesion length and ratio of in-stent restenosis were not significantly different between the HD and non-HD groups (*p* = 0.56 and *p* = 0.8, respectively). No particular DCB device was associated with HD status (*p* = 0.2).

### 3.2. Freedom from CDTLR, Mortality, Major Amputation

**Figure 1:**
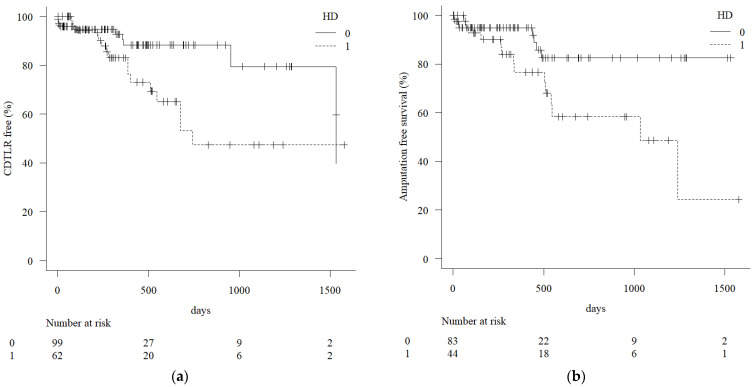
Kaplan–Meyer curves of the lesions of the patients with and without chronic kidney disease on hemodialysis. The uninterrupted line (HD 0 in the graph field) is the curve of those not on hemodialysis, and the interrupted line (HD 1 in the graph field) is of those on hemodialysis. The HD group consistently exhibits worse free-from-CDTLR survival (**a**) (*p* = 0.02) and amputation-free survival (**b**) (*p* = 0.01).

The Kaplan–Meier survival curve (Figure 1) illustrates the cumulative incidence of CDTLR-free survival (left) and amputation-free survival (right) over time (in days) for the two groups: the non-HD group (solid line) and the HD group (interrupted line). The CDTLR was analyzed for l61 lesions. The non-HD group demonstrated a significantly higher CDTLR-free rate compared to the HD group throughout the follow-up period. At around 1500 days, the CDTLR-free rate remains above 80% for the non-HD group, while the HD group’s rate declines to approximately 45%. The HD group exhibits a steeper decline in CDTLR-free survival within the first 500 days, indicating that this group experiences earlier CDTLR compared to the non-HD group. The table below the graph shows the number of patients at risk at various time points. The initial sample size was 99 for the non-HD group and 62 for the HD group. Over time, the number of individuals at risk decreases, reflecting censoring and events. The plus (+) symbols on the curves indicate censored data points, where patients were lost to follow-up or did not experience the event by the end of the study. In summary, the non-HD group has a significantly better CDTLR-free survival rate compared to the HD group, suggesting that HD is associated with a higher risk of CDTLR occurrence over time. This difference appears to be particularly pronounced in the early follow-up period.

Amputation-free survival was analyzed for 127 patients. The Kaplan–Meier curve of amputation-free survival declines to nearly 20% at the end of the follow-up period. As only 2 amputations were observed during this study period, the decline in AFS in the HD group largely reflects poor overall survival rather than limb amputation. The reasons for mortality in this study were not related to FP EVT using DCBs, but were not well documented in the study data.

According to the results of the log-rank test to analyze the statistical significance of the difference in the duration of freedom from CDTLR, the median duration of freedom from CDTLR was 744 days in the HD group and 1533 days in the non-HD group (*p* = 0.02). The amputation-free survival was also worse in the HD group (*p* = 0.01) (Figure 1). The all-cause mortality was 13 (30.0%) for the HD group and 7 (8.4%) for the non-HD group (*p* = 0.004). One patient from both the HD group and the non-HD group underwent major limb amputations (*p* = 1).

The multivariate analysis, using a binary logistic regression analysis, showed HD as a significant predictor of CDTLR with an odds ratio of 4.48 (*p* = 0.03) (Table 2). History of smoking is another statistically significant predictor of CDTLR, with an odds ratio of 3.63 (*p* = 0.05). To estimate the relationship between lesion severity and CDTLR, factors such as R4 to 6, calcification, ISR, and occluded lesions were examined through multivariate analysis using binary logistic regression. However, none of these severity-related factors demonstrated a statistically significant association with CDTLR.

## 4. Discussion

A previous study on the management of FP lesions reported that balloon dilation without a drug coating was associated with worse outcomes in dialysis patients [8]. Recent evidence suggests that a DCB outperforms balloon dilation without a drug coating in the treatment of LEADs involving the FP region [15]. This study was conducted to test the impact of HD on FP EVT using DCBs, and the significant negative impact of HD on the primary outcome was observed based on the Kaplan–Meier analysis (Figure 1). The multivariate Cox regression analysis also identifies HD as a significant predictor of CDTLR with an odds ratio of 4.48. Among the study cohort, 11 patients (2 patients with two lesions in the HD group and 9 patients with nine lesions in the non-HD group) had a follow-up period of less than 30 days, which might be considered too short for inclusion in this study. However, since follow-up naturally ends at the time of an outcome event, excluding patients solely based on short follow-up duration may not be reasonable. Among these 11 patients, one HD patient died and one non-HD patient underwent amputation. Given these events, we considered it appropriate to include these patients in the analysis. Furthermore, sensitivity analysis showed that excluding these patients resulted in an even greater negative impact of HD. After excluding these patients, the duration of freedom from CD-TLR remained significantly shorter in the HD group (median: 1032 days) compared to the non-HD group (median: 1515 days; *p* = 0.01), and multivariate Cox regression analysis showed that statistical significance remained unchanged across all demographic factors, with an odds ratio of 5.74 for HD (*p* = 0.03) in particular.

Target lesion calcification is reported to be characteristic of HD patients, and this calcification is regarded to be related to a worse outcome after EVT for FP lesions [16], and a worse TASC II classification, a higher rate of chronic total occlusion, a longer lesion length, and a higher rate of severe calcification in HD patients have been reported [17]. In this study, the calcification of the target lesion was not found to be a significant factor for the primary outcome in the multivariate analysis (Table 2). This statistical insignificance of the relationship between lesion calcification and CDTLR in this study cohort was consistent with the previous literature because PACSS grade 4 lesions, which are reported to have worse prognosis than PACSS grade 1 to 3 lesions [13], were not treated with DCBs in this study cohort. In addition, the lesion length and occlusion ratio were similar in both groups, which can be explained by a possible interrelationship between TASC II classification, chronic total occlusion, lesion length, and severe calcification [17]. The findings above suggest that the overall target lesion severity, including the calcification, legion length, and occlusion, was not significantly different between the two groups in this study. The multivariate Cox regression analysis was unable to identify calcification, occlusion, ISR, or R4-6 as being related to CDTLR (Table 2). Lesion severity did not differ significantly between the HD and non-HD groups, nor was it significantly associated with CDTLR. These findings suggest that HD is an independent risk factor for CDTLR following DCB treatment of FP lesions.

In the HD group, the patients were significantly younger, and CAD was more prevalent (Table 1). These findings were congruent with previous studies [18,19], suggesting that HD-requiring patients have accelerated vascular calcification and atherosclerosis. The higher mortality in the HD group can also be explained by the higher prevalence of CAD in this group. The association between a major amputation and HD-requiring CKD was not significant due to the limited incidence of this event. Patients in the HD group had a shorter duration of freedom from CDTLR and were more likely to have CDTLR, but the underlying causes remain undetermined. Thus, further data collection and analysis are warranted to enhance the results of EVT for FP LEADs in HD-requiring CKD patients.

The limitations of this study include a relatively small sample size, the use of three different types of DCBs, and the retrospective nature of this study. First, the sample size is sufficient for showing statistical significance of HD on CDTLR, but the low event rate of amputation in this study, compounded by the small sample size, limits our ability to draw definitive conclusions, especially for amputation-free survival. Second, in this study cohort, three DCBs, namely the IN.PACT, Ranger, and Lutonix, which differ in their paclitaxel concentration, were selected based on physician preference. This variability may have influenced the results, as high-dose DCBs are reported to outperform low-dose DCBs in some studies [20,21]. Third, in this study, we could not define HD as a causal relationship because of the retrospective nature of this study. Lastly, the lack of outflow data is a critical limitation of this study, as the outflow below the knee lesion is the significant prognostic factor for the FP EVT [22], and HD patients have significantly worse vessel outflow [23]. This potentially significant confounding factor could not be analyzed in this study due to a lack of data.

## 5. Conclusions

This study demonstrated that patients with CKD requiring HD experienced poorer outcomes following femoropopliteal EVT using a DCB, with an odds ratio of 4.48 for CDTLR. Further investigations are warranted to validate these findings in larger cohorts, incorporating a comprehensive analysis of confounding variables and detailed patient backgrounds to better understand the underlying mechanisms contributing to worse outcomes in HD patients.

## Figures and Tables

**Table 1:** Characteristics of the patients and lesions.

Total Patients *n* = 127		HD group *n* = 44	non-HD group *n* = 83	*p* value
Median Age		70.5	72.5	<0.001
Male Sex		35 (79.5%)	53 (63.9%)	0.04
HT		32 (72.7%)	55 (66.3%)	0.84
DL		15 (34.1%)	28 (33.7%)	0.23
DM		34 (77.3%)	51 (61.4%)	0.03
CAD		27 (61.4%)	17 (20.5%)	<0.001
CVD		11 (25.0%)	28 (33.7%)	0.42
Smoking history		32 (72.7%)	64 (77.1%)	0.53
Total Target Lesions *n* = 161		HD group *n* = 62	non-HD group *n* = 99	*p* value
DCB used	IN. PACT	19	42	0.31
	Lutonix	17	24	
	Ranger	26	33	
Rutherford’s category	2, 3	11 (17.7%)	46 (46.5%)	<0.001
	4, 5, 6	51 (82.3%)	53 (53.5%)	
Stenosis		57 (91.9%)	78 (78.8%)	0.03
Occlusion		5 (8.1%)	21 (21.2%)	
lesion length (mm)		107.3±57.9	112.7±56.8	0.52
Calcification		54 (87.1%)	68 (68.9%)	0.01
ISR		4 (6.5%)	9 (9.1%)	0.77

HT, hypertension; DL, dyslipidemia; DM, diabetes mellitus; CAD, coronary artery disease; CVD cerebrovascular disease; HD, hemodialysis; DCB, drug-coated balloon; ISR, in-stent restenosis.

**Table 2:** Multivariate Cox regression analysis of the background factors affecting clinically driven target lesion revascularization.

Factors	Hazard Ratio	95% Confidence Interval			*p* Value
DL	0.66	0.23	-	1.85	0.43
DM	0.56	0.19	-	1.60	0.28
HD	3.18	1.05	-	9.68	0.04
HT	0.43	0.17	-	1.12	0.09
smoking	3.50	0.99	-	12.39	0.05
ISR	0.61	0.07	-	5.23	0.65
occlusion	1.65	0.45	-	5.98	0.45
R2.3	0.61	0.20	-	1.91	0.4
R4.6	NA	NA	-	NA	NA
CAD	1.06	0.42	-	2.67	0.9
CVD	1.00	0.36	-	2.79	1
calcification	0.60	0.18	-	1.98	0.4

CAD, coronary artery disease; CVD cerebrovascular disease; DLD, dyslipidemia; DM, diabetes mellitus; HD, hemodialysis; HTN, hypertension; ISR, in-stent restenosis.

## Data Availability

The data supporting the findings of this study are not publicly available, as approval for data sharing was not sought from the ethics committee prior to the study.
